# Dysbiotic alteration in the fecal microbiota of patients with polycystic ovary syndrome

**DOI:** 10.1128/spectrum.04291-23

**Published:** 2024-07-11

**Authors:** Ke Chen, Huafeng Geng, Cong Ye, Junbao Liu

**Affiliations:** 1 Department of Gynecology, China-Japan Union Hospital of Jilin University, Changchun, Jilin, China; Jilin University, Changchun, Jilin, China

**Keywords:** polycystic ovary syndrome, gut microbiota, metabolism, *Bifidobacterium*, *Bacteroides*

## Abstract

Polycystic ovary syndrome (PCOS) is a common disease associated with high androgen and infertility. The gut microbiota plays an important role in metabolic diseases including obesity, hyperglycemia, and fatty liver. Although the gut microbiota has been associated with PCOS, little is known about the gut microbial structure and function in individuals with PCOS from Northeast China. In this study, 17 PCOS individuals and 17 age-matched healthy individuals were recruited for community structure and function analysis of the gut microbiota. The results showed that PCOS individuals have reduced diversity and richness of the gut microbiota compared with healthy individuals. Beta diversity analysis showed that the community structure of the gut microbiota of individuals with PCOS was significantly separated from healthy individuals. At the phylum level, PCOS individuals have reduced *Firmicutes* and *Bacteroidota* and increased *Actinobacteriota* and *Proteobacteria* compared with healthy individuals. At the family and genus levels, the composition of the gut microbiota between PCOS patients and healthy individuals was also significantly different. In addition, PICRUSt2 showed that individuals with PCOS have different microbial functions in the gut compared with healthy individuals. We finally confirmed that *Bifidobacterium* was enriched in the fecal samples of PCOS patients, while other 11 genera including *Bacteroides*, *UCG_002*, *Eubacterium__coprostanoligenes_group_unclassified*, *Dialister*, *Firmicutes_unclassified*, *Ruminococcus*, *Alistipes*, *Christensenellaceae_R_7_group*, *Clostridia_UCG_014_unclassified*, *Roseburia*, and *Lachnospiraceae_unclassified* were depleted compared with healthy individuals. These results indicate that individuals with PCOS have altered community structure and functions of the gut microbiota, which suggests that targeting the gut microbiota might be a potential strategy for PCOS intervention.

**IMPORTANCE:**

Gut microbiota plays a critical role in the development of PCOS. There is a complex and close interaction between PCOS and gut microbiota. The relationship between the pathogenesis and pathophysiological processes of PCOS and the structure and function of the gut microbiota needs further investigation.

## INTRODUCTION

Polycystic ovary syndrome (PCOS) is one of the most common gynecological endocrine disorders with a very complex etiology and pathogenesis (1). The prevalence of PCOS is as high as 5% to 10% in women of reproductive age (2). It accounts for 50% to 70% of anovulatory infertility (3). According to epidemiological surveys in China, the prevalence of PCOS among Han Chinese women is as high as 5.6% (4). PCOS is characterized by biochemical or clinical manifestations of hyperandrogenism, polycystic ovarian changes, and persistent anovulation and may be associated with abdominal obesity, insulin resistance, impaired glucose metabolism, and dyslipidemia (5). It is the most common cause of menstrual disorders and the main cause of anovulatory infertility in women of childbearing age (6). In addition, PCOS can increase the risk of gestational diabetes, coronary heart disease, endometrial cancer, and other diseases in the long run (7). Not only does PCOS have a high prevalence, but its management and treatment costs are enormous (8). According to the Food and Drug Administration (FDA), the cost of managing and treating PCOS in the United States exceeded $4 billion in 2004, not including the cost of managing and treating the long-term complications of PCOS (9). PCOS is a heavy burden for a country with a large population like China. Therefore, early detection, early prevention, early and reasonable intervention, and avoidance of long-term complications are very important (10). Currently, most scholars believe that polycystic ovary syndrome is a disease controlled by multiple genes and triggered by multiple factors (11). The pathogenesis of PCOS is unclear, and many patients have poor clinical outcomes. Therefore, further in-depth studies to elucidate the pathogenesis of PCOS are crucial.

There are 10 to 100 trillion bacteria living in the human intestine, belonging to hundreds to thousands of different species (12). Their number is 10 times more than the number of human cells and 150–200 times more genes than the number of human genes (13). Under normal conditions, intestinal bacteria form a mutually beneficial symbiotic relationship with both other bacteria and the host in the intestine, creating a relatively complex and complementary environment known as the microecosystem (14). These symbiotic microorganisms in the human gut are a large “undiscovered” organ of the human body, and some scholars believe that the human body itself is a symbiotic organism formed by a combination of human cells and bacteria (15). This micro-ecosystem provides humans with rich nutrients and important micronutrients, promotes the maturation and differentiation of the immune system, and plays a role in many important physiological functions of the human body (16). They provide a large amount of nutrients to the body: they also defend the intestinal mucosa against pathogenic bacteria to protect the health of the host (17). Normal adult intestinal flora is in a complex dynamic balance in the human intestine (18). In recent years, with the advancement of technologies such as second- and third-generation sequencing, validated with the help of sterile animals, it has been found that gut microbes are directly associated with a large number of diseases (19). Gut microbes even play an etiological role in many diseases such as obesity, diabetes, atherosclerosis, and inflammatory bowel disease (20). It has been reported in the literature that the gut microbiota is a central regulator of host metabolism, which regulates many metabolic processes in the host, including energy homeostasis, glucose metabolism, and lipid metabolism (21). Microbial imbalances, sometimes referred to as ecological disorders, are associated with metabolic perturbations. Several studies have shown a causal relationship between microbial function and metabolic perturbations (22). Therefore, treatments targeting intestinal microbes have been shown to improve metabolic function in humans.

The relevance of gut microbes to metabolic diseases has become a research hotspot, bringing many new ideas to the etiology and pathological mechanisms of PCOS. Many studies have shown that the gut microbiota plays an important role in the development and progression of PCOS (23, 24)Pearce et al. proposed in 2012 that long-term poor dietary habits lead to intestinal microbial dysbiosis, and disordered intestinal microorganisms lead to impaired intestinal mucosal barrier function, causing lipopolysaccharide (LPS) from Gram-negative bacteria to enter the body circulation. LPS entering the body circulation activates the immune system, causing chronic inflammation, and long-term chronic inflammation interferes with insulin receptor function, leading to increased insulin secretion by pancreatic B cells. As a result, serum insulin levels increase compensatorily, causing insulin resistance, which promotes ovarian androgen synthesis. This leads to an increase in the synthesis of androgens in the ovary and interferes with the normal development of follicles, which eventually leads to the development of polycystic ovary syndrome (25). Recent studies have shown that PCOS leads to characteristic changes in the gut microbiota. At the same time, targeted changes to gut microbes can affect PCOS symptoms (26). Gut microbes may influence PCOS symptoms through metabolites, such as bile acids, short chain fatty acid (SCFA)s, and LPS. These potential mechanisms of action provide a theoretical basis for PCOS therapies targeting gut microbes (27). One study analyzed the gut microbiology of PCOS patients and PCOS animal models and found significant differences in gut microbial composition between the PCOS group and the normal control group. The decrease in diversity and the change in the abundance of specific bacterial genera suggested a possible correlation between gut microbes and the development of PCOS (28). While confirming the presence of intestinal microbiota disorders in PCOS patients, Torres et al. suggested that these changes may be associated with hyperandrogenemia. The intestinal microbiota acts as an “endocrine organ” for maintaining human health. The gut microbiota affects the reproductive endocrine system by interacting with estrogens, androgens, and insulin (29). Typical features of PCOS include abnormal sex hormone levels, insulin resistance, polycystic ovarian changes, and chronic subclinical inflammation (30). Dysbiosis of the gut microbiota is involved in endotoxemia, SCFA production, bile acid metabolism, and abnormal secretion of brain intestinal peptides. The above physiological and pathological processes are associated with the manifestations of PCOS, such as hyperandrogenism, insulin resistance, chronic inflammatory response, and abnormal levels of brain-gut peptides (31). Therefore, the gut microbiota may influence follicular development, sex hormone, and metabolic levels through hyperandrogenism, insulin resistance, chronic inflammation, and brain-gut axis, and participate in the pathogenesis of PCOS. The modulation of gut microbiota to improve the metabolism of PCOS may be one of the potential options for the future treatment of PCOS, but the exact mechanisms remain to be explored in depth. In conclusion, there is a complex and close interaction between PCOS and gut microbiota. The relationship between the pathogenesis and pathophysiological processes of PCOS and the structure and function of the gut microbiota needs further investigation.

## RESULTS

### Descriptive statistics

We recruited 17 PCOS patients and 17 healthy individuals in the current study. The average age of PCOS individuals is 25.53 years, while the average age of healthy individuals is 23.87 years, but no significance was identified (Table 1). Consistently, no significance was detected in height between PCOS and healthy individuals (Table 1). However, PCOS patients have higher body weights than healthy individuals (*P* = 0.0490, Table 1). Similarly, PCOS patients have higher body mass index (BMI) than healthy individuals (*P* = 0.0665, Table 1). The serum hormone levels of PCOS patients, including luteinizing hormone, follicle-stimulating hormone (FSH), prolactin, estradiol (E2), testosterone (T), fasting blood glucose, total cholesterol, triglyceride, low-density lipoprotein, high-density lipoprotein, and fasting insulin, were also detected for PCOS diagnosis (Table 1).

**TABLE 1:** Characteristics of study participants^*a*^

Characteristics	Healthy controls (*n* = 17)	Patients with PCOS (*n* = 17)	*P* value
Demographics			
Mean age (year), mean ± SD	23.87 ± 2.642	25.53 ± 4.185	0.3824
Height, cm, mean ± SD	162.2 ± 4.570	160.7 ± 4.370	0.2631
Weight, kg, mean ± SD	54.82 ± 4.549	62.18 ± 11.52	0.0490
BMI, mean ± SD	20.83 ± 1.454	24.21 ± 5.201	0.0665
Disease characteristics			
LH	NA	8.362 ± 3.998	
FSH	NA	5.235 ± 0.8645	
PRL	NA	249.9 ± 199.9	
E2	NA	99.36 ± 67.85	
T	NA	1.271 ± 0.3099	
FBG	NA	5.126 ± 0.6149	
TCHO	NA	5.197 ± 0.9635	
TG	NA	1.86 ± 1.657	
LDL	NA	3.027 ± 0.6704	
HDL	NA	1.338 ± 0.3529	
FSlns	NA	16.42 ± 11.59	

^*a*^ LH, luteinizing hormone; PRL, prolactin; E2, estradiol; T, testosterone; FBG, fasting blood glucose; TCHO, total cholesterol; TG, triglyceride; LDL, low-density lipoprotein; HDL, high-density lipoprotein; FSlns, fasting insulin.

### Alpha diversity of the gut microbiota

**Fig 1:**
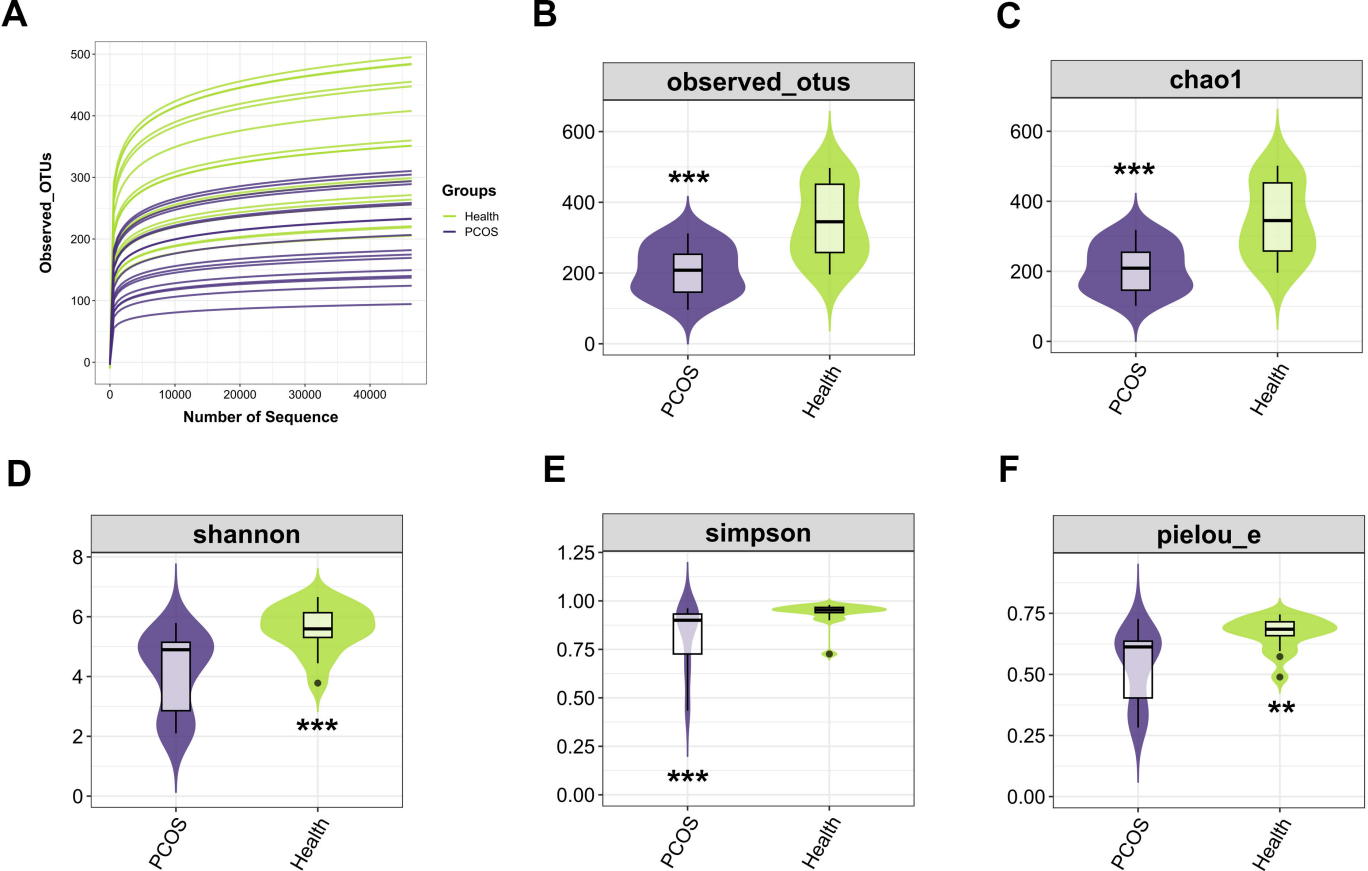
Alpha diversity of the gut microbiota. (**A and B**) The PCOS groups had reduced levels of observed OTUs significantly compared with the Health group (Mann-Whitney *U*-test, *n* = 17). (**C**) Chao1 results showed that PCOS could change the microbial numbers in the fecal samples, and it was decreased observably in the Shannon index (Mann-Whitney *U*-test, *n* = 17). (**D and E**) Shannon and Simpson indicated that patients with PCOS had reduced microbial diversity compared with healthy individuals (Mann-Whitney *U*-test, *n* = 17). (**F**) Pielou_e index (Mann-Whitney *U*-test, *n* = 17). **P < 0.01 and ***P < 0.001 indicate significance.

Alpha diversity analysis was performed to reveal microbial community richness, diversity, and evenness by observed operational taxonomic units (OTUs), Shannon, Chao1, Simpson, and pielou_e. We first found that individuals with PCOS had reduced observed OTUs compared with those from the Health group (*P*﹤0.001, Mann-Whitney *U*-test, Fig. 1A and B). A reduced Chao1 index was also observed in patients with PCOS compared with those in the Health group (*P*﹤0.001, Mann-Whitney *U*-test, Fig. 1C), indicating that patients with PCOS had fewer microbial numbers in the fecal samples. We next identified that patients with PCOS had reduced microbial diversity characterized by the decreases in Shannon and Simpson indices in the fecal samples of individuals with PCOS compared with individuals in the Health group (*P*﹤0.001, Mann-Whitney *U*-test, Fig. 1D and E). In addition, the decrease in fecal microbial diversity in the PCOS patients was confirmed by the pielou_e index (*P*﹤0.001, Mann-Whitney *U*-test, Fig. 1F). Altogether, these results indicate that patients with PCOS had reduced alpha diversity in the gut microbiota.

### Microbial structure by β diversity analysis

**Fig 2:**
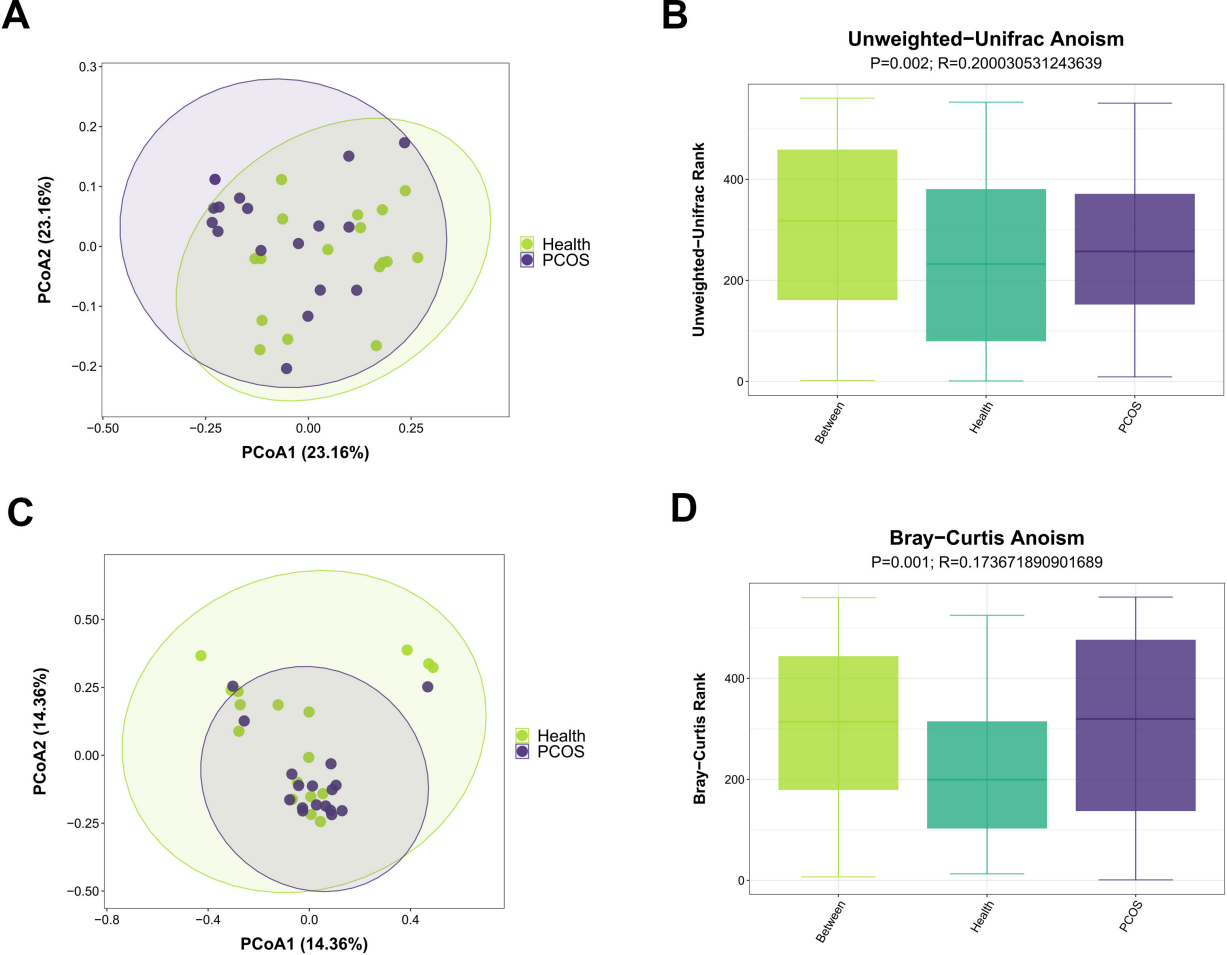
Microbial structure by β diversity analysis. (**A**) Gut microbial structures of PCOS patients and healthy individuals were analyzed by PCoA based on unweighted UniFrac distance (*n* = 17). (**B**) Similarity analysis indicated that the microbial community was different between the PCOS and Health groups (*P* = 0.002, *R* = 0.2000, ANOSIM, *n* = 17). (**C–D**) Based on Bray-Curtis distance, PCoA results showed that the intestinal microbial community structure of PCOS patients was different from individuals from the Health group (*P* = 0.001, *R* = 0.1736, ANOSIM, *n* = 17).

Principal coordinates analysis (PCoA) was performed for β diversity analysis. The overall gut community of PCOS patients was separated from that of the Health group as indicated by PCoA based on unweighted UniFrac distance (Fig. 2A). Next, the statistical significance was tested by analysis of similarities (ANOSIM), and the result showed that microbial community was significantly different between PCOS patients and healthy individuals (*P* = 0.002, *R* = 0.2000, ANOSIM, Fig. 2B). To confirm these results, PCoA based on Bray-Curtis distance was performed. Similarly, the gut microbial community of PCOS patients was different from those in the Health group (*P* = 0.001, *R* = 0.1736, ANOSIM, Fig. 2C and D). Together, these results indicate that patients with PCOS had different gut microbial structures compared with healthy individuals.

### Fecal microbial community composition

**Fig 3:**
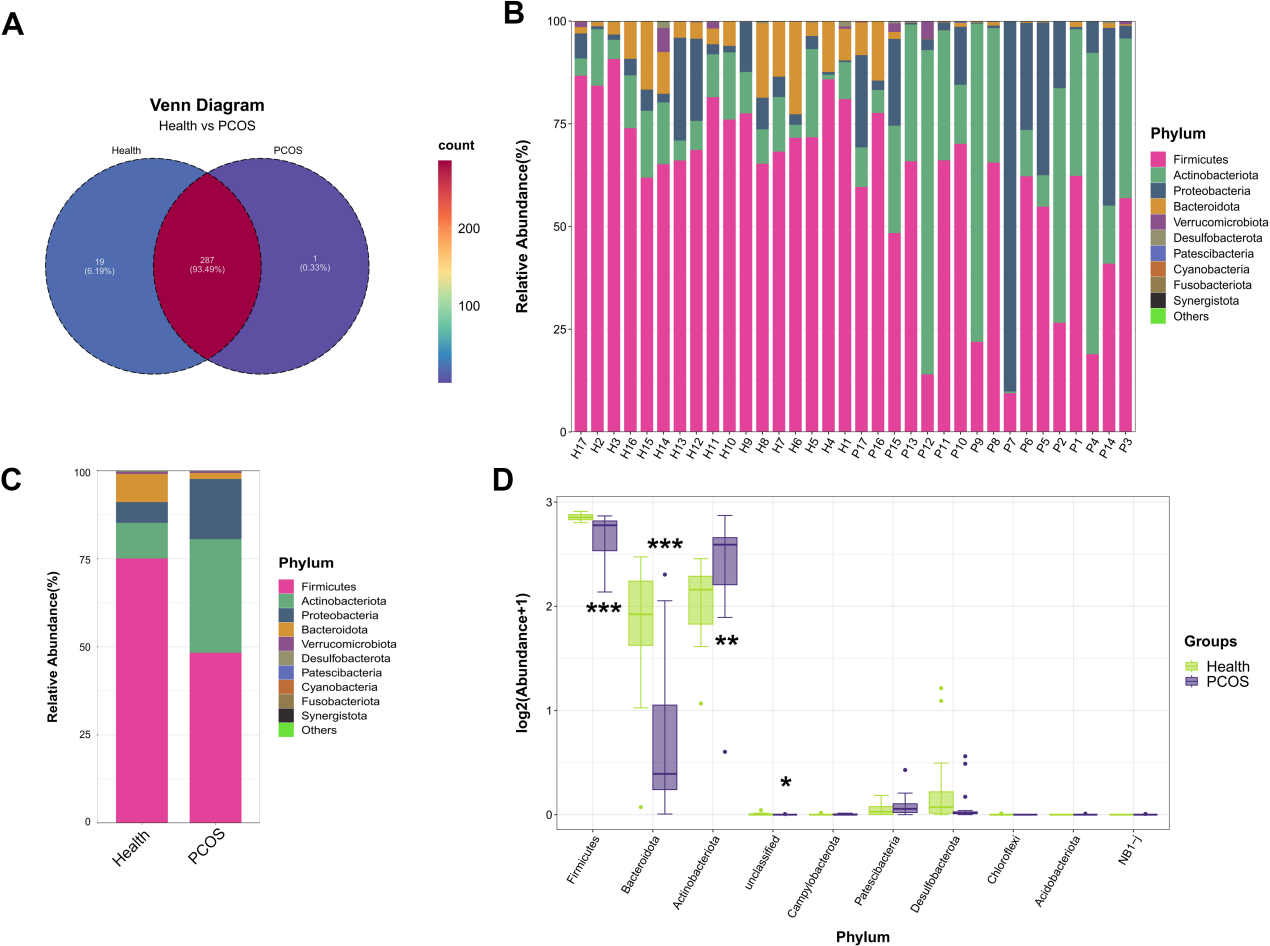
Fecal microbial community composition at the phylum level. (**A**) Venn diagram displayed the common OTUs and the different OTUs between PCOS patients and healthy controls (*n* = 17). (**B–C**) The composition of the gut microbiota of PCOS patients and healthy individuals at the phylum level (*n* = 17). (**D**) Comparison of intestinal microbiota abundance of PCOS patients and Health group at the phylum level (Mann-Whitney *U*-test, *n* = 17). *P < 0.05, **P < 0.01, ***P < 0.001 indicate significance.

To reveal the microbial composition of the gut microbiota in all 34 fecal samples, the Venn diagram was first performed at the OTU level. A total of 307 OTUs were identified in the gut microbiota of the PCOS patients and healthy individuals (Fig. 3A). Among the identified microbes, 19 OTUs (6.19%) existed only in the healthy individuals, and 1 OUT (0.33%) was observed only in the PCOS patients, while 287 OTUs (93.49%) were commonly found in both PCOS patients and healthy individuals (Fig. 3A). At the phylum level, microbial composition between the PCOS patients and healthy individuals was different as indicated by reduced relative abundances of *Firmicutes* and *Bacteroidota,* and enhanced *Proteobacteria* and *Actinobacteriota* were observed in PCOS patients compared with healthy individuals (Fig. 3B and C). To confirm these results, the Mann-Whitney *U*-test was performed followed by false discovery rate (FDR) correction. The result showed that lower relative abundances of *Firmicutes* and *Bacteroidota* were identified in PCOS patients than in healthy individuals (*p_fdr_*﹤0.001, Mann-Whitney *U*-test, Fig. 3D), while a higher relative abundance of *Actinobacteriota* was detected in patients with PCOS than those in the Health group (*p_fdr_*﹤0.01, Mann-Whitney *U*-test, Fig. 3D).

**Fig 4:**
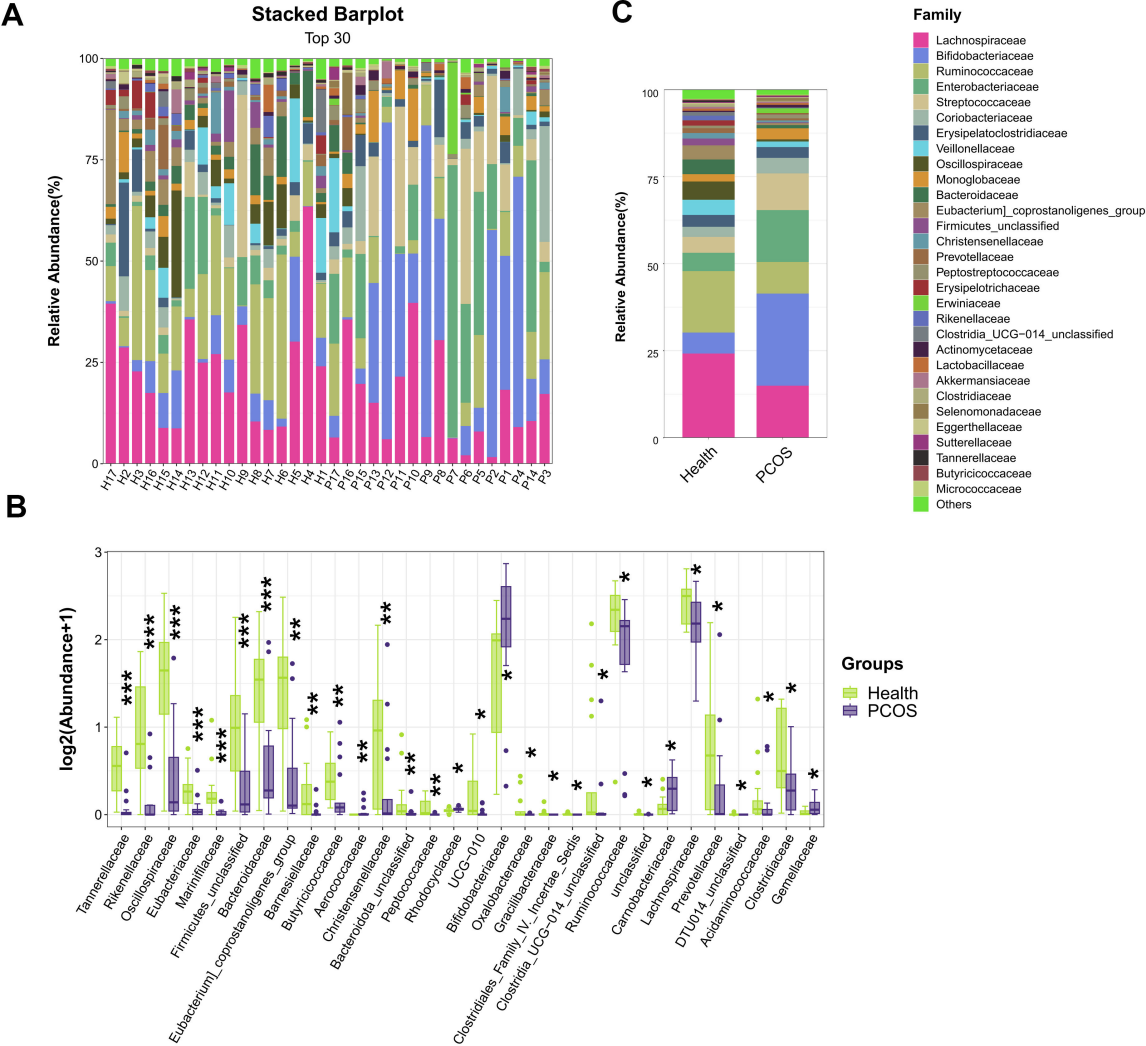
Fecal microbial community composition at the family level. (**A and B**) The difference in gut microbiota between PCOS patients and healthy controls was analyzed at the family level (*n* = 17). (**C**) At the family level, the significantly different microbes of PCOS patients and healthy controls were analyzed (Mann-Whitney *U*-test, *n* = 17). *P < 0.05, **P < 0.01, ***P < 0.001 indicate significance.

At the family level, higher relative abundances of *Bifidobacteriaceae*, *Enterobacteriaceae*, *Streptococcaceae*, *Coriobacteriaceae*, *Monoglobaceae*, and *Erwiniaceae* were observed in the fecal samples of PCOS patients than in healthy individuals (Fig. 4A and B), while lower relative abundances of *Lachnospiraceae*, *Ruminococcaceae*, *Veillonellaceae*, *Eubacterium]_coprostanoligenes_group*, *Bacteroidaceae*, *Erysipelotrichaceae,* and *Rikenellaceae* were detected in the fecal samples of PCOS patients than those in the Health group (Fig. 4A and B). Next, the top 30 different families were identified by the Mann-Whitney *U*-test. The result showed that the relative abundances of *Bifidobacteriaceae* (*p_fdr_* = 0.0150), *Carnobacteriaceae* (*p_fdr_* = 0.0280), *Rhodocyclaceae* (*p_fdr_* = 0.0113), and *Aerococcaceae* (*p_fdr_* = 0.0082) were greater in the PCOS group than in the Health group (Fig. 4C), while *Tannerellaceae* (*p_fdr_*﹤0.001), *Rikenellaceae* (*p_fdr_*﹤0.001), *Oscillospiraceae* (*p_fdr_*﹤0.001), *Eubacteriaceae* (*p_fdr_*﹤0.001), *Marinifilaceae* (*p_fdr_*﹤0.001), *Firmicutes_unclassified* (*p_fdr_*﹤0.001), *Bacteroidaceae* (*p_fdr_*﹤0.001), *Eubacterium]_coprostanoligenes_group* (*p_fdr_* = 0.0018), *Barnesiellaceae* (*p_fdr_* = 0.002), *Butyricicoccaceae* (*p_fdr_* = 0.0068), *Christensenellaceae* (*p_fdr_* = 0.0089), *Bacteroidota_unclassified* (*p_fdr_* = 0.0094), *Peptococcaceae* (*p_fdr_* = 0.01), *UCG-010* (*p_fdr_* = 0.014)*, Oxalobacteraceae* (*p_fdr_* = 0.015), *Gracilibacteraceae* (*p_fdr_* = 0.018), *Clostridiales_Family_IV._Incertae_Sedis* (*p_fdr_* = 0.018), *Clostridia_UCG-014_unclassified* (*p_fdr_* = 0.024), *Ruminococcaceae* (*p_fdr_* = 0.026), *Lachnospiraceae* (*p_fdr_* = 0.031), *Prevotellaceae* (*p_fdr_* = 0.033), *DTU014_unclassified* (*p_fdr_* = 0. 036), *Acidaminococcaceae* (*p_fdr_* = 0. 0.037), *Clostridiaceae* (*p_fdr_* = 0. 0.044), and *Gemellaceae* (*p_fdr_* = 0.047) were greater in the Health group than in the PCOS group (Fig. 4C).

**Fig 5:**
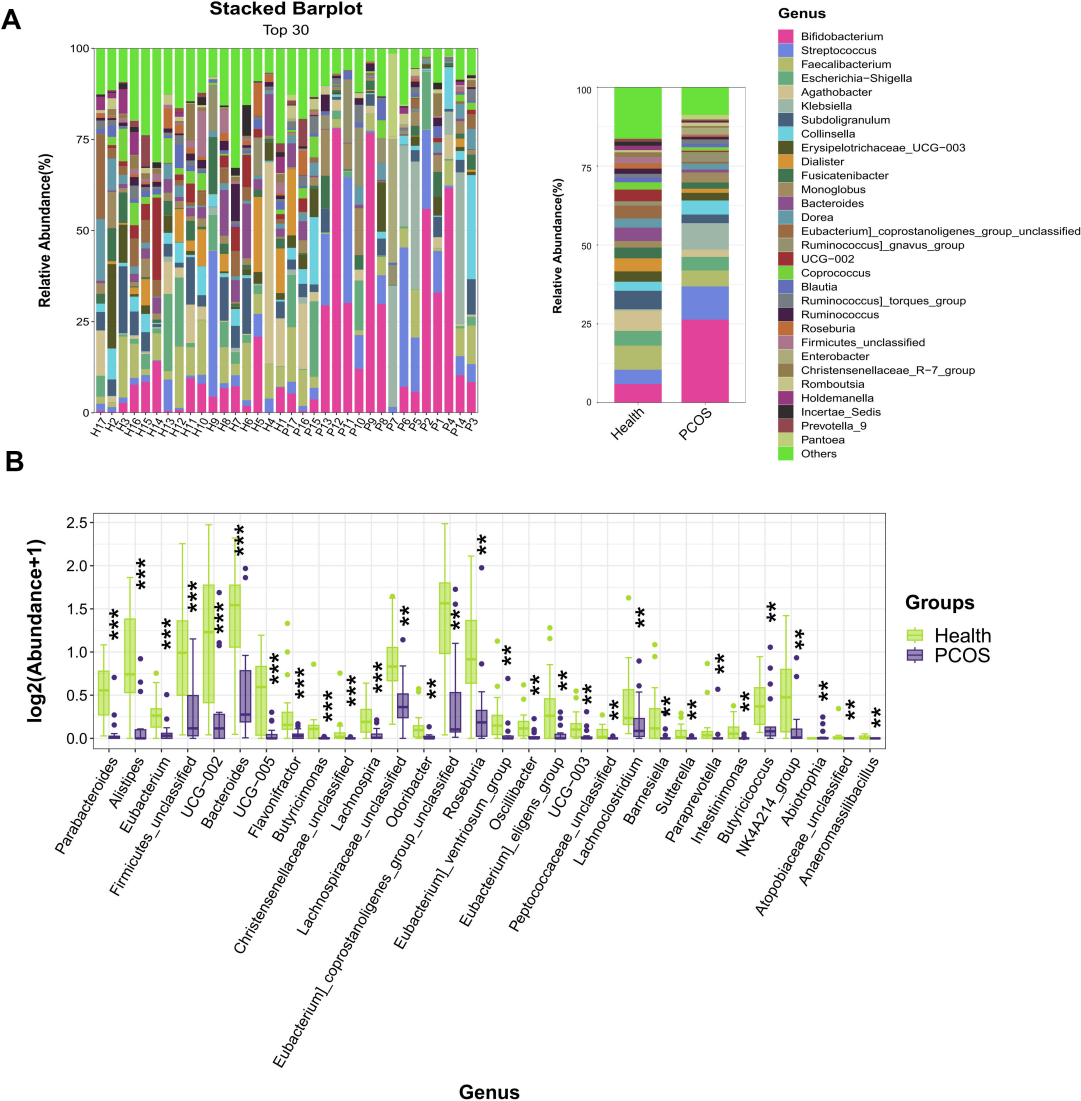
Fecal microbial community composition at the genus level. (**A**) The composition of the gut microbiota of PCOS patients and healthy individuals at the genus level (*n* = 17). (**B**) The top 30 different genera were analyzed by Mann-Whitney U-test, *n* = 17. *P < 0.05, **P < 0.01, ***P < 0.001 indicate significance.

At the genus level, higher relative abundances of *Bifidobacterium*, *Streptococcus*, *Klebsiella*, *Collinsella*, *Monoglobus,* and *Ruminococcus]_gnavus_group* were observed in the fecal samples of the PCOS patients than in healthy individuals (Fig. 5A and B), while lower *Faecalibacterium*, *Agathobacter*, *Subdoligranulum*, *Dialister*, *Bacteroides*, *Dorea*, *UCG−002,* and *Coprococcus* were detected in the PCOS group than the Health group (Fig. 5A and B). Furthermore, the top 30 different genera were also confirmed by the Mann-Whitney U-test. The results showed that *Parabacteroides* (*p_fdr_* ﹤0.001), *Alistipes* (*p_fdr_* ﹤0.001), *Eubacterium* (*p_fdr_* ﹤0.001), *Firmicutes_unclassified* (*p_fdr_* ﹤0.001), *UCG-002* (*p_fdr_* ﹤0.001), *Bacteroides* (*p_fdr_* ﹤0.001), *UCG-005* (*p_fdr_* ﹤0.001), *Flavonifractor* (*p_fdr_* ﹤0.001), *Butyricimonas* (*p_fdr_* ﹤0.001), *Christensenellaceae_unclassified* (*p_fdr_* ﹤0.001), *Lachnospira* (*p_fdr_* = 0.001), *Lachnospiraceae_unclassified* (*p_fdr_* = 0.0011), *Odoribacter* (*p_fdr_* = 0.0016), *Eubacterium]_coprostanoligenes_group_unclassified* (*p_fdr_* = 0.0018), *Roseburia* (*p_fdr_* = 0.0018), *Eubacterium]_ventriosum_group* (*p_fdr_* = 0.0021), *Oscillibacter* (*p_fdr_* = 0.0027), *Eubacterium]_eligens_group* (*p_fdr_* = 0.0045), *UCG-003* (*p_fdr_* = 0.0054), *Peptococcaceae_unclassified* (*p_fdr_* = 0.0059), *Lachnoclostridium* (*p_fdr_* = 0.0062), *Barnesiella* (*p_fdr_* = 0.0062), *Sutterella* (*p_fdr_* = 0.0066), *Paraprevotella* (*p_fdr_* = 0.0069), *Intestinimonas* (*p_fdr_* = 0.0069), *Butyricicoccus* (*p_fdr_* = 0.0076), *NK4A214_group* (*p_fdr_* = 0.0082), *Abiotrophia* (*p_fdr_* = 0.0082), and *Atopobiaceae_unclassified* (*p_fdr_* = 0.0082) were depleted in the PCOS group compared with the Health group (Fig. 5C). Collectively, these results indicate that patients with PCOS had changed gut microbial profiles.

### Microbial function prediction

**Fig 6:**
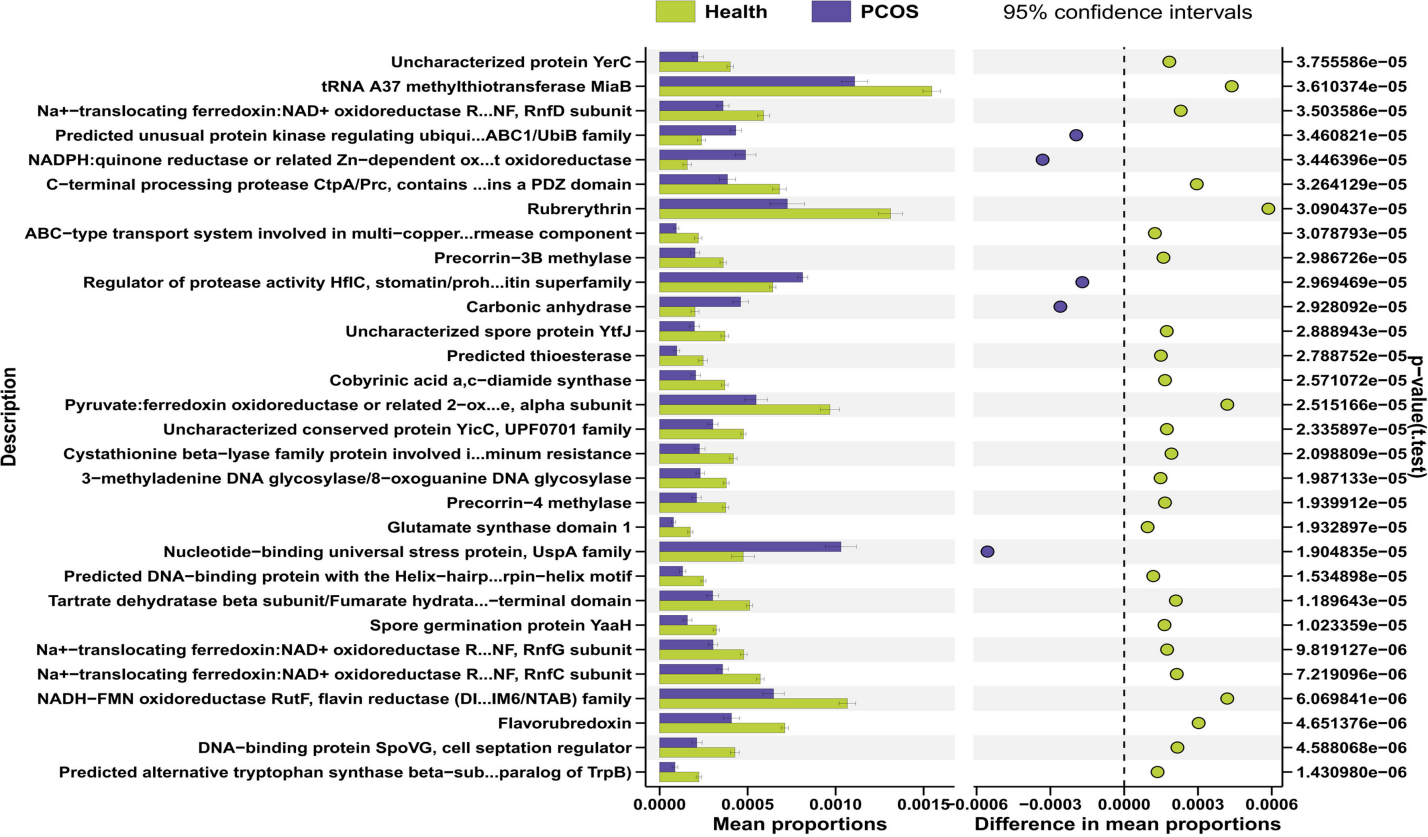
Microbial function prediction by PICRUSt2.

We next investigated the TOP 30 gut microbial functions of fecal samples using PICRUSt2. The results showed that microbial functions associated with Regulator of protease activity HflC, stomatin/prohibitin superfamily (*p_fdr_* ﹤0.001), Nucleotide-binding universal stress protein, UspA family (*p_fdr_* ﹤0.001), NADPH:quinone reductase or related Zn-dependent oxidoreductase (*p_fdr_* ﹤0.001), Carbonic anhydrase (*p_fdr_* ﹤0.001), and Predicted unusual protein kinase regulating ubiquinone biosynthesis, AarF/ABC1/UbiB family (*p_fdr_* ﹤0.001) were enriched in the gut microbiota of the PCOS patients compared with healthy individuals (Fig. 6), while other 25 microbial functions, including Glutamate synthase domain 1 (*p_fdr_* ﹤0.001), Rubrerythrin (*p_fdr_* ﹤0.001), Flavorubredoxin (*p_fdr_* ﹤0.001), Predicted alternative tryptophan synthase beta-subunit (paralog of TrpB) (*p_fdr_* ﹤0.001), Predicted thioesterase, tRNA A37 methylthiotransferase MiaB (*p_fdr_* ﹤0.001), 3−methyladenine DNA glycosylase/8−oxoguanine DNA glycosylase (*p_fdr_* ﹤0.001), and Spore germination protein YaaH (*p_fdr_* ﹤0.001), were depleted in the gut microbiota of the PCOS patients compared with healthy individuals (Fig. 6). Together, these results suggest that PCOS patients had altered gut microbial functions.

### PCOS patient-associated bacterial genera

**Fig 7:**
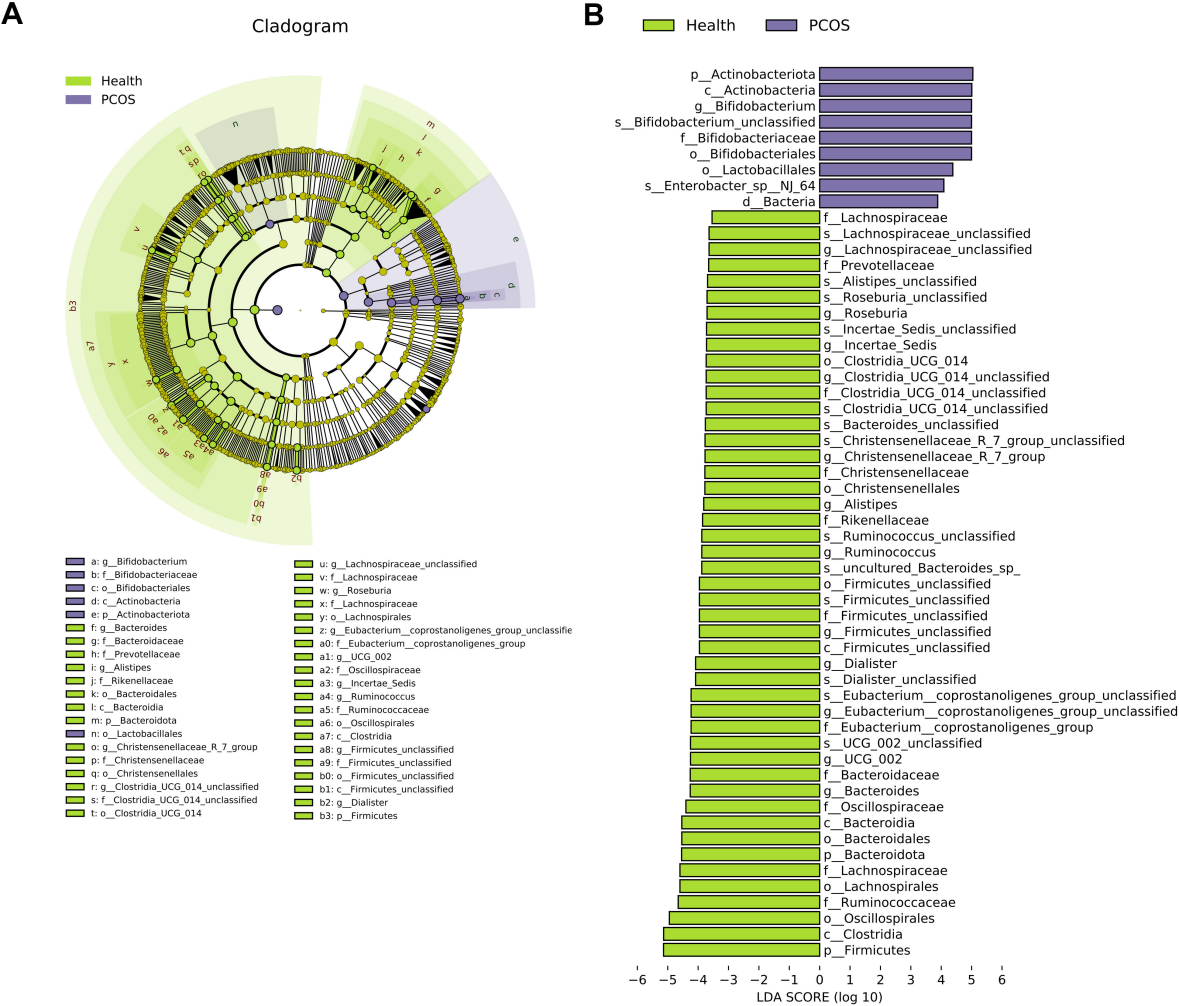
PCOS patient-associated microbial species. (A and B) Different bacterial genera were identified by LEfSe (LDA score (log 10) ＞4).

Furthermore, linear discriminant analysis effect size (LEfSe) was performed to confirm the different genera in the gut microbiota between PCOS patients and healthy individuals (LDA SCORE (log 10) ＞ 4). The results showed that *Bifidobacterium* was enriched in the fecal samples of PCOS patients compared with healthy individuals (Fig. 7A and B), while *Bacteroides*, *UCG_002*, *Eubacterium__coprostanoligenes_group_unclassified*, *Dialister*, *Firmicutes_unclassified*, *Ruminococcus*, *Alistipes*, *Christensenellaceae_R_7_group*, *Clostridia_UCG_014_unclassified*, *Roseburia*, and *Lachnospiraceae_unclassified* were depleted in the fecal samples of the PCOS patients compared with healthy individuals (Fig. 7A and B). These results indicate that PCOS individuals had altered gut microbial communities.

## DISCUSSION

PCOS is a metabolic disease characterized by higher androgen and is often accompanied by metabolic disorders, such as insulin tolerance, hyperglycemia, and obesity, which can lead to infertility in severe cases. Gut microbiota has been reported to be related to the development of many diseases, such as mastitis, diabetes, and arthritis. Studies found that the gut microbiota was associated with sex hormone concentrations in both human and animal models. Additionally, regulation of the gut microbiota by fecal microbiota transplantation, probiotics, and prebiotics could alleviate PCOS (32, 33). These results suggest that the gut microbiota plays a significant role in PCOS pathogenesis. In the current study, we found that PCOS individuals from Northeast China have changed the community structure and functions of the gut microbiota. Similar to our finding, previous studies found that individuals with PCOS had reduced alpha diversity and changed beta diversity in the gut microbiota compared with healthy controls (34–36).

For the community composition, we identified that *Bifidobacterium* was enriched in the gut of PCOS patients. Although *Bifidobacterium* has been known as a widely used probiotic for disease intervention, including obesity and asthma (37, 38), the protective effects of *Bifidobacterium* have been reported to be associated with bile acid metabolism (38). However, bile acid metabolic disorder has been shown to play a significant role in PCOS pathogenesis (34). In detail, gut microbiota-derived glycodeoxycholic acid and tauroursodeoxycholic acid promote the production of interleukin-22 by intestinal group 3 innate lymphoid cells and subsequently limit PCOS development. Gut *Bacteroides vulgatus* contained bile salt hydrolase (*bsh*) genes that could metabolize glycodeoxycholic acid and tauroursodeoxycholic acid and thus induce PCOS-like symptoms (34). Notably, *Bifidobacterium* is known to contain *bsh* genes, which endows *Bifidobacterium* to promote PCOS development but needs further confirmation (39).

Previously, studies reported that *Bacteroides* were enriched in the gut of PCOS patients, especially *Bacteroides vulgatus* (34, 36). The gap between our study and previous studies might be the differences in geographical location and dietary patterns of PCOS patients. Indeed, Paris et al. found that different dietary patterns could alter the composition and function of the gut microbiota and subsequently influence PCOS development in mice (40). These findings suggest that regulation of the gut microbiota by diet nutrition may be a potential target for PCOS intervention.

Our study revealed that many short-chain fatty acid-produced bacteria, including *Dialister*, *Ruminococcus*, *Alistipes*, *Christensenellaceae_R_7_group*, *Clostridia_UCG_014_unclassified*, *Roseburia,* and *Lachnospiraceae_unclassified*, were depleted in the gut microbiota of PCOS individuals. Li et al. found that increased fecal *Ruminococcus* by tempol treatment could alleviate PCOS caused by Dehydroepiandrosterone (DHEA), which was associated with the production of stachyose (41). Although no evidence showed that the relative abundance of *Alistipes* is associated with PCOS, many studies found that *Alistipes* abundance was significantly depleted in individuals with obesity (42). *Dialister* and *Alistipes* were found to produce succinate, acetate, and propionate, which have been reported to alleviate obesity, insulin tolerance, and hyperglycemia (43, 44). *Roseburia* was reported to produce butyrate and alleviate gut dysbiosis-associated disease (45, 46). These results suggest that SCFA might play a significant role in regulating the pathogenesis of PCOS but needs further investigation.

In summary, our research indicates that individuals with PCOS have altered structure, composition, and functions of the gut microbiota, which confirms the essential role of the gut microbiota in PCOS and provides new insight for PCOS intervention by targeting the gut microbiota, as well as acts as a basis for other metabolic diseases.

## MATERIALS AND METHODS

### Human sample collection

The study was approved by the Ethics Committee of Jilin University according to the Council for International Organizations of Medical Sciences. All participants were recruited from the China-Japan Union Hospital between December 2022 and June 2023. We recruited 17 individuals with PCOS and 17 healthy individuals of Chinese ancestry. Written informed consent was obtained from all participants. Women with PCOS were diagnosed according to the 2003 Rotterdam criteria and previous study (34). All individuals with PCOS were first-visit patients and had not received PCOS-related treatment. The healthy individuals were from the general community and had regular menstrual cycles, normal ovarian morphology, and normal levels of hormones. Height, body weight, and BMI were calculated. Peripheral blood samples were collected from all subjects during days 2–4 of spontaneous cycles after an overnight fast. Fecal samples from PCOS and healthy controls were collected for 16S rRNA sequence.

### Hormone-level determination

Levels of serum hormone, including FSH and luteinizing hormone, were tested by radioimmunoassays as previously described (34). The levels of estradiol, testosterone, androstenedione, and DHEA sulfate were measured using liquid chromatography–mass spectrometry (Sciex Triple Quad 6500+). The levels of fasting serum glucose, serum insulin, triglycerides, total cholesterol, high-density lipoprotein cholesterol, and low-density lipoprotein cholesterol were measured using an autoanalyzer (Beckman Coulter AU5800).

### Fecal DNA extraction

DNA from different samples was extracted using the CTAB according to the manufacturer’s instructions (QIAGEN). The reagent that was designed to uncover DNA from trace amounts of sample is effective for the preparation of the DNA of most bacteria. Nuclear-free water was used for blank. The total DNA was eluted in 50 µL of Elution buffer and stored at −80°C until measurement in the PCR by LC-Bio Technology Co., Ltd (Hang Zhou, Zhejiang Province, China).

### 16S ribosomal RNA (16S rRNA) gene sequencing

The primers used in the current study are 341F (5′-CCTACGGGNGGCWGCAG-3′) and 805R (5′-GACTACHVGGGTATCTAATCC-3′). The 5′ ends of the primers were tagged with specific barcodes per sample and sequencing universal primers. PCR amplification was performed in a total volume of 25 µL reaction mixture containing 25 ng of template DNA, 12.5 µL PCR Premix, 2.5 µL of each primer, and PCR-grade water to adjust the volume. The PCR conditions to amplify the prokaryotic 16S fragments consisted of an initial denaturation at 98℃ for 30 seconds, 32cycles of denaturation at 98℃ for 10 seconds, annealing at 54℃ for 30 seconds, extension at 72℃ for 45 seconds, and then final extension at 72℃ for 10 minutes. The PCR products were confirmed with 2% agarose gel electrophoresis. Throughout the DNA extraction process, ultrapure water, instead of a sample solution, was used to exclude the possibility of false-positive PCR results as a negative control. The PCR products were purified by AMPure XT beads (Beckman Coulter Genomics, Danvers, MA, USA) and quantified by Qubit (Invitrogen, USA). The amplicon pools were prepared for sequencing, and the size and quantity of the amplicon library were assessed on Agilent 2100 Bioanalyzer (Agilent, USA) and with the Library Quantification Kit for Illumina (Kapa Biosciences, Woburn, MA, USA), respectively. The libraries were sequenced on the NovaSeq PE250 platform.

### Statistical analyses and visualization

Samples were sequenced on an Illumina NovaSeq platform according to the manufacturer’s recommendations, provided by LC-Bio. Paired-end reads were assigned to samples based on their unique barcode and truncated by cutting off the barcode and primer sequence. Paired-end reads were merged using FLASH. Quality filtering on the raw reads was performed under specific filtering conditions to obtain high-quality clean tags according to the fqtrim (v0.94). Chimeric sequences were filtered using Vsearch software (v2.3.4). After dereplication using DADA2, we obtained a feature table and feature sequence. Alpha diversity and beta diversity were calculated by normalizing to the same sequences randomly. Then according to the SILVA (release 138) classifier, feature abundance was normalized using the relative abundance of each sample. Alpha diversity is applied in analyzing the complexity of species diversity for a sample through five indices, including Chao1, Observed species, Goods coverage, Shannon, and Simpson, and all these indices in our samples were calculated with QIIME2. Beta diversity was calculated by QIIME2, and the graphs were drawn by R package. Blast was used for sequence alignment, and the feature sequences were annotated with the SILVA database for each representative sequence. Other diagrams were implemented using the R package (v3.5.2).

## Data Availability

The raw data supporting the conclusion of this article will be made available by the authors, without undue reservation, to any qualified researcher. Data has also been deposited under BioProject number PRJNA981428.
